# Developing a virtual reality for people with dementia in nursing homes based on their psychological needs: a feasibility study

**DOI:** 10.1186/s12877-021-02125-w

**Published:** 2021-03-07

**Authors:** Jung-Hee Kim, Seonmin Park, Hyeongji Lim

**Affiliations:** College of Nursing, The Catholic University of Korea, 222 Banpo-daero Seocho-gu, Seoul, 06591 Korea

**Keywords:** Dementia, Virtual reality, Virtual reality intervention, Cognitive dysfunction

## Abstract

**Background:**

The purpose of this study was (1) to develop a virtual reality (VR) intervention program based on the psychological needs of patients residing in nursing facilities in South Korea to alleviate their behavioral and psychological symptoms and (2) to confirm the possibility of utilizing VR in patients with dementia.

**Methods:**

In the first phase, patients with dementia residing in nursing homes and experiencing behavioral and psychological symptoms were recruited. Surveys and questionnaires were used to identify activities that alleviated the behavioral and psychological symptoms of dementia (BPSD) among the patients. These activities were classified into five types of psychological needs. In the second phase, a fully immersive, interactive, easy-to-use VR platform was developed that reflected these psychological needs. Patients with dementia experienced the VR content. The researchers assessed the level of the participants’ immersion, preference, and interaction with the VR using a 5-point Likert scale.

**Results:**

In the feasibility test, 10 nursing home residents were recruited. The mean immersion score was 4.93 ± 0.16 points, the mean preference score was 4.35 ± 0.41 points, and the mean interaction score was 3.84 ± 0.43 points using a 5-point Likert scale. Higher mean scores indicated a more positive outcome. Six of the 10 participants required assistance while using the VR. The mean VR experience duration was 10.00 ± 3.46 min.

**Conclusions:**

The VR-based intervention program that was developed to reduce BPSD was feasible for the participants and provided them with a high degree of satisfaction and immersion. Furthermore, this study also confirmed the convenience and safety of the program. These findings support the potential use of VR-based BPSD intervention programs to treat patients with dementia.

**Supplementary Information:**

The online version contains supplementary material available at 10.1186/s12877-021-02125-w.

## Background

The number of people with dementia is rapidly increasing due to the aging of the global population. According to Alzheimer’s Disease International, in 2018, there were 50 million individuals with dementia globally, which represents a 6% increase from 2015 [1]. In South Korea, the prevalence of dementia in persons 65 years and older, as of 2018, was 10.1%, and the number has continued to increase [2].

Patients with dementia experience cognitive dysfunction and various behavioral and psychological symptoms, such as nervousness, depression, psychosis, yelling, and violence [3, 4]. Due to their loss of social and communication skills and limitations in their ability to undertake physical activity, patients with dementia experience issues in their relationships with others. This can exacerbate negative emotions and behavioral and psychological issues in older patients with dementia [5, 6]. The behavioral and psychological symptoms of dementia (BPSD) are one of the most significant reasons why those with dementia receive early admission into nursing facilities [7]. The early institutionalization into a nursing home leads to higher social costs and a lower quality of life for patients with dementia and their families [8].

Interventions that decrease BPSD include non-pharmacological interventions that focus primarily on reducing and removing the psychosocial or environmental risk factors associated with behavioral and psychological symptoms [9] and therapeutic approaches, such as recall therapy, horticultural therapy, music therapy, art therapy, animal-assisted therapy, and physical exercise [10–12]. However, it is difficult to apply existing non-pharmacological interventions to patients with dementia and decreased cognitive function. The application of these interventions is restricted due to a lack of trained professionals [13]. To overcome these restrictions, multisensory virtual reality (VR) platforms may be used to improve cognitive function and rehabilitation in patients with dementia, as VR platforms are advanced, adaptable, and easy-to-use [14–18]. VR refers to technologies that allow people to experience realistic situations or environments that are difficult to experience in reality [19, 20]. In particular, immersive VR can be used to focus on certain sensory stimuli and has been reported to facilitate positive emotions and improvements in their emotional state, interpersonal interactions, and communication [21–23].

The motivation to use VR may differ between individuals based on individual interests and preferences, aside from maladjustment to VR [13]. The psychological needs of people with dementia include comfort, identity, attachment, occupation, and inclusion [24]. While studies on patient-centered dementia care have considered these needs [25–28], very few studies have developed activities or interventions for dementia patients that consider their psychological needs.

This study involved three phases. The objective of the first phase was to confirm the activities that alleviate BPSD and classify these activities based on the patients’ psychological needs. The objective of the second phase was to develop VR platform environments based on the patients’ psychological needs and test whether it alleviates BPSD. The objective of the final phase was to evaluate the participants’ VR immersion, preferences and interactions with VR, and tolerance for VR.

## Methods

### Ethics approval and consent to participate

The project was approved by the Institutional Review Board of the Catholic University of Korea (MC18QNSI0055). Before participation, the researchers obtained written informed consent from each patient and the patient’s legal guardian or representative. The participants were recruited by recruitment notices posted in facilities after obtaining approval from the institution’s managers. Once identified, the mental health expert manager spoke with the patients to see whether they would agree to be contacted for participation in the study. Once agreed, we contacted the participants’ legal guardians or representatives to recruit the participants and obtain consent to participate. The participants were assured of the anonymity and confidentiality of all data collected and given an opportunity to ask questions. To overcome the short-term memory problems and variable capacity, the participants were informed of their right to withdraw at any stage from the study without giving a reason.

### Phase 1: psychological needs to alleviate symptoms

This phase identified the psychological needs that alleviate the symptoms of the patients using polling surveys and questionnaires. The data source was a large-scale research project focused on BPSD to develop an intervention program for improving quality of life. In this study, the convenience sampling method was used, and details of the sampling process have been previously reported in Park et al. [29]. Patients with dementia were recruited from six nursing homes located in two cities. Participants’ preferred environment for intervention development was investigated. Their quality of life and symptoms of psychological impairment were measured to verify the effect of the intervention program. In this study, the data selection process was as follows. Among the recruited 325 participants, participants who reported behavioral and psychological symptoms were selected. Of these participants, only those who answered the questions addressing the activities that alleviated their BPSD were included. Based on this, 103 participants were included in the study, and 222 participants were excluded from the study.

A survey question addressed the activities that alleviated BPSD in patients: “When you feel agitation, aggression, psychosis, depression, and apathy, what activities make you feel better or alleviate your BPSD?” If the participant could not answer the question, the question was completed by asking the care staff who had been caring for the participant for at least 4 weeks (Additional file 1). The care staff responded with their observations on behalf of the patients. The answers were sorted and compiled into response categories and subcategories. Additionally, qualitative data from the participants’ narratives were analyzed thematically based on the psychological needs of patients with dementia [30–32].

Based on the five psychological needs of dementia patients [30], the activities were classified into the character-strengthening aspects of comfort (being free from distress and pain, experiencing reduced anxiety by receiving tenderness and friendliness, and feeling soothed), identity (having a sense of self, knowing details of their life history, knowing who one is in relation to significant others, and having a sense of continuity with the past), attachment (feeling security and safety, and trust), occupation (having a purpose in life and being empowered to have an impact), and inclusion (a feeling of belonging, being encouraged to interact with the social environment physically and emotionally). Two independent researchers (A and B) classified the alleviating activities based on the psychological needs of patients with dementia.

### Phase 2: development of a VR intervention

The VR was designed using the analysis of psychological needs that alleviate BPSD, which was explored in Phase 1. The VR project was then implemented through collaboration with an external company that had the resources, experience, and time to meet the study requirements.

#### Design of VR and participants’ actions

The VR content was developed to fulfill the five psychological needs (comfort, identity, attachment, occupation, and inclusion) of the patients with dementia to strengthen the participants’ personality. The following sections detail the content and interactive factors of the VR experience (Table 1).

**Table 1:** Summary of intervention program of virtual reality that addresses psychological needs

Title	Places	Psychological needs	Contents	Multimedia	Interactive factors	Session length (min)
Train of memories	Train station	Occupation Inclusion	Admiring the scenery at the train station Boarding the train and choosing a destination	Sound of the train	Giving train tickets to station attendant	1
Street of memories	Elementary school Alleyway My homely house (City)	Identity Inclusion Occupation Attachment	Looking around the playgrounds and classrooms of elementary schools Playing with friends in the neighborhood alley Coming home and playing with puppies Receiving gifts and looking at family photos	Sound of puppies barking Family photo	Erasing graffiti on the classroom blackboard Playing slap-match Playing with puppies Receiving family gifts	4
Nostalgic youth	Theater Coffee house Market My homely house (City)	Identity Inclusion Attachment	Admiring the surroundings and entering the theater Watching an old film (Korea news) Drinking tea in a coffee house Visiting the market Coming home and playing with puppies Receiving gifts and looking at family photos	The bell rang at dawn (1972) New invention (1981) Olympic closing ceremony (1988) The sound of water boiling in kettle Market noise Sound of puppies barking Family photo	Video selection Drinking black herbal tea Buying radish Playing with puppies Receiving family gifts	6.5
Homely hometown	Valley Field of reed My homely house (Countryside)	Identity Comfort Attachment	Flowing water in the valley Walking in a field of reed Returning to the country home and lighting a fire in the furnace Coming back to the room and viewing family photos	The sound of flowing valleys Water splashing The sound of the wind passing through the reeds Crackling sound of fire Family photo	Dipping one’s hand in the valley water Stroking the reeds Putting a kindling in the furnace Receiving family photos	3.5
Where I want to go	Orchards Namiseom Island The sea at night Crocks of condiments Juknokwon Bamboo Garden	Comfort	Walking through the orchards Taking a walk in the Namiseom Island Watching lighthouses, waves, and stars in the night sea Looking at the rain falling on crocks Walking through the bamboo forest while listening to Daegeum playing	Sound of cicadas Sound of the wind Sound of the waves Sound of the rain Sound of the wind, Korean traditional music		10

The VR was designed to meet the comfort needs of the participants, including physical touch (patting a friend on the shoulder), place of memory, forming a quiet environment, and walking. Identity needs were met by visiting family and viewing family pictures, while the inclusion needs were reflected in conversation, being with someone, positive expression, and words/expressions of acknowledgment. Attachment needs included hanging out with friends and family, and the occupation needs included selecting a destination that the patients wanted to visit, erasing graffiti, going grocery shopping, playing with dogs, and putting logs into a fireplace.

With the theme of “Train of memories,” this study implemented VR in 15 different places using four categories. The participant could choose the content via hand movements, and the content was categorized into “Streets of memory,” “Nostalgic youth,” “Homely hometown,” and “Where I want to go.” In the “Train of memories,” the participant could choose their destination by handing a train ticket to the train station attendant and could enjoy the scenery of the old train station. In the “Streets of memory,” the program depicted elementary schools of the past, neighborhood alleys, and old houses, and the participants experienced slap-match games in alleyways with friends, erasing graffiti on school blackboards, playing with puppies at home, and seeing family photos. “Nostalgic youth” included watching an old film at a theater, drinking tea in a traditional coffee house, and going home after shopping for groceries at a traditional market. “Homely hometown” allowed the participants to experience interactions such as playing in a river, stroking reed grass while walking in a breezy field, and placing kindling in a fireplace in a rural home. “Where I want to go” included visual and auditory stimuli, such as orchards, Namiseom Island, the sea at night, observing the rain, and the Jungnogwon Bamboo Garden. There were no intellectual or functional demands required during interactions with the virtual environment.

#### Multimedia technological design and implementation

Through collaboration with the external company, we created an interactive VR program for relieving BPSD. This intervention provided a fully immersive audio-visual experience of the different virtual environments. Sound, music, photos, and movies were added, in addition to anecdotes and verbal cues. To meet the psychological needs of older people with dementia, the content consisted of different environments, music, and natural sounds suitable for fostering the positive emotions of South Koreans.

**Fig. 1:**
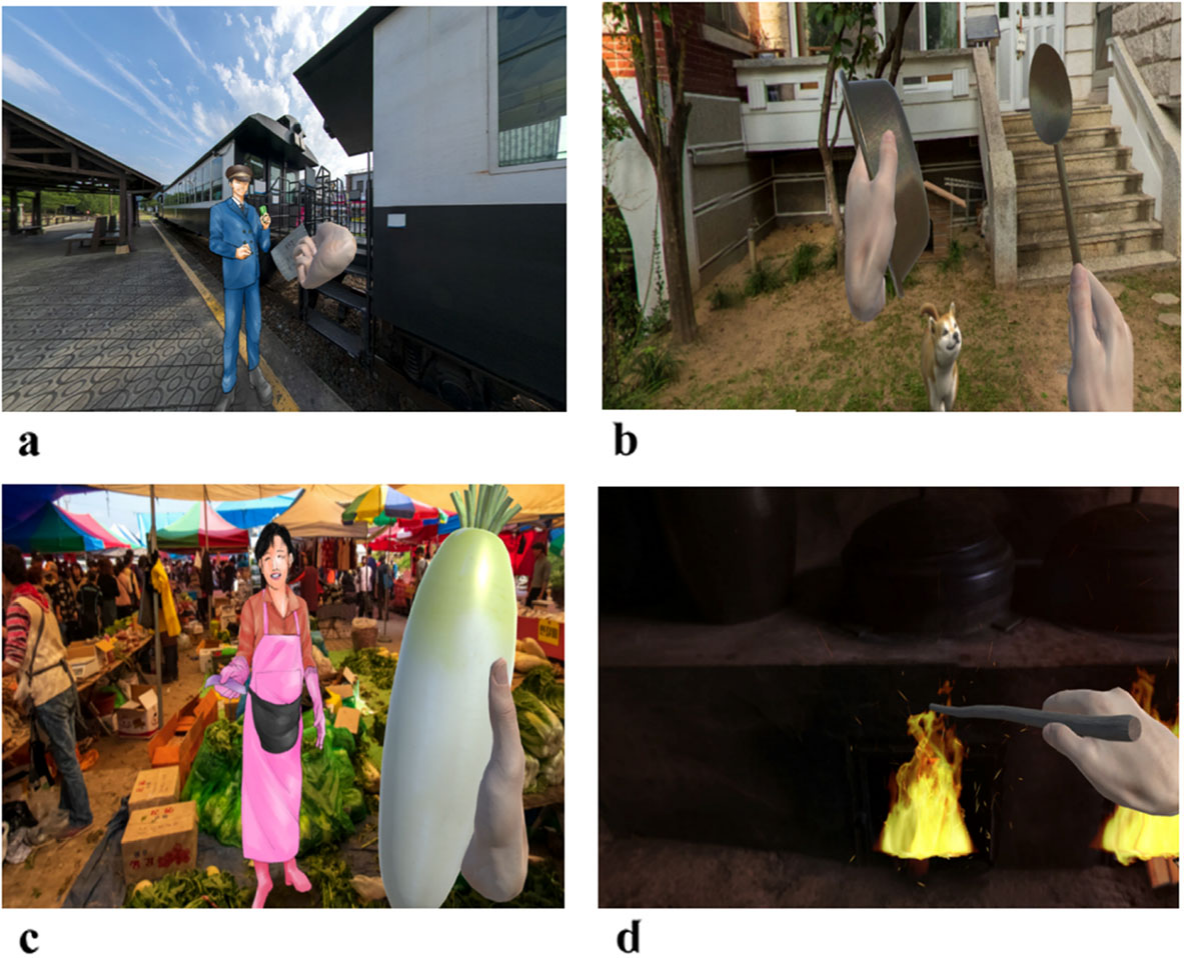
Some screenshots from virtual reality interactions in this study**:** a) in “Train of memories,” a participant handing a train ticket to the train station attendant, b) in “Streets of memory,” a participant playing with puppies while playing bowls at home, c) in “Nostalgic youth,” a participant shopping for groceries at a traditional market, d) in “Homely hometown,” a participant placing kindling in a fireplace in a rural home

The VR program was implemented using 360-degree video viewing, a 360-degree camera recording of the natural environment, editing, and graphics production, which maximized the reality and immersion. The interactions were implemented by attaching a leap motion to the Head Mounted Display (HMD; Samsung HMD Odyssey Windows Mixed Reality Headset plus Wireless controller), allowing for easy recognition of the participants’ hand movements. The seasons and various animated objects could be manipulated through leap motion sensors (LM-010), allowing the participants to interact with the scene through hand and arm movements. Particular movements moved animated objects, such as train tickets, blackboard erasers, slap cards, puppies, teacups, radishes, valley water, reeds, and firewood. The participants did not need to use joysticks or keyboards (Fig. 1).

The platform allowed for overall easy solo use at home or with some assistance, thereby ensuring the platform’s safety. The participants were seated next to their caregiver or a research assistant throughout the VR session; postural demands were reduced by the participants remaining in their chair during the VR exercises.

### Phase 3: feasibility test

#### Procedures

In Phase 3, the participants were also recruited through recruitment notices in residential and daycare facilities for older people. The participants were selected based on the following criteria: (1) they were a resident or daycare visitor with dementia, (2) they were aged 65 years or above, (3) they had a Mini Mental Status Examination-Korean version (MMSE-K) score of 15 or higher, (4) they had a Clinical Dementia Rating of 0.5 or higher, and (5) they understood the research process and agreed to participate. The exclusion criteria consisted of motor dysfunction due to cerebral infarction, other mental disorders, neurological disorders, and metabolic disorders. A total of 10 participants were recruited.

The VR-based intervention programs were provided to the participants in the program rooms of the institutions in 1–2 sessions for 20–30 min each. The intervention times were customized for the participants; lunch hours, visiting hours, and napping hours were avoided.

The participants’ age, MMSE-K scores, and Activities of Daily Life (ADL) scores were obtained from their medical records. The MMSE-K scores ranged from 0 to 30, with higher scores indicating better cognition and a score below 24 indicating cognitive impairment [33].

The participants used the VR at their own pace after receiving guidance from the trained researchers. They were guided to specific places they wanted to visit and experienced two to three VR places. To assess the immersion, preference, and interaction during the VR experience, the researchers asked the participants to respond to three questions relating to immersion, preferences, and degree of interaction in their VR experiences on a 5-point Likert scale (1 = “Very poor,” 2 = “Poor,” 3 = “Fair,” 4 = “Good,” 5 = “Excellent”) in every place they chose. Higher mean scores indicated a more positive experience, and the answers to these questions were obtained through the structured questionnaire.

To identify the participants’ tolerance for VR, the researchers recorded the need for assistance, duration of the VR experience, positive or negative experiences, and VR sickness (Additional file 2). Positive or negative experiences were measured by their visual alertness and verbal engagement. The following negative behaviors were recorded: complaining, agitation, wandering, hitting, grabbing, pushing, throwing objects, biting, hurting self or others, tearing objects or destroying property, and making physical/verbal sexual advances. The following positive behaviors were recorded: remaining seated and still, being focused, being calm, smiling, and communicating verbally or non-verbally. Two researchers observed and recorded the participants’ responses, and disagreements between their ratings were resolved by discussion and reaching a consensus. For the statistical analyses of the data, SPSS Statistics 27.0 was used, and these included frequency and descriptive analyses, in which the means, standard deviations, medians, and interquartile ranges (IQR) were analyzed.

## Results

### Activities that alleviate BPSD

Activities that alleviated BPSD among the patients were characterized and classified into five types of psychological needs. The need for “comfort” was rated highest in terms of providing relief from BPSD, with 36.9% out of a total of 103 patients making this assessment, followed by the need for “identity” (28.2%), “inclusion” (24.3%), “attachment” (10.7%), and “occupation” (6.8%). Activities related to the need for comfort included walking, eating snacks, physical contact (such as hugging and holding hands), and creating a quiet environment. Activities related to the need for identity included religious activities and visits from guardians and family members. Activities related to the need for inclusion involved receiving positive support from others. Activities related to the need for attachment included checking objects (checking their clothes and placing a name sticker on personal closets). Activities related to the need for occupation included reading, solving puzzles, and playing traditional South Korean card games (Table 2).

**Table 2:** Classification of psychological needs based on relieving activities for BPSD (*N* = 103)

Psychological needs	Relieving activities for BPSD	N (%)
Comfort	▪ Taking a walk, moving (from own room to living room), reading magazines (picture books) ▪ Taking medicine, receiving nutritional shots, getting one’s prescription filled, sensory stimuli (listening to music, applying liquid painkiller), coffee, snack ▪ Creating a quiet environment, shutting the door, staying in one’s room and coming out to the living room to exercise when no one is there, sitting in the toilet, taking a bath for more than an hour ▪ Doing what one wants when immediate request is granted ▪ Hugging, holding hands, physical contact, looking at the visitor log and confirming family’s visit, listening to what others are saying, being told warmly that they need to leave tomorrow	38 (36.9)
Identity	▪ Reading the bible, hymnbook, listening to pastor’s sermons, worshipping, thinking of God, always having prayer beads by the bedside ▪ Calling home, visit by caregiver, visit by younger sibling, visit by sons, visit by daughters, visit by grandchildren, photos with the family, visit by family, chatting with a daughter, receiving attention, told by son that he would call, told that their children are coming ▪ Isolated from daughter-in-law, refraining from visiting children	29 (28.2)
Attachment	▪ Checking for one’s own belongings, checking for clothes, wearing familiar clothes, placing name tags in individual closets, touching individual belongings with permission from elderly with dementia, receiving what one wants, bags, radio (Far East Broadcasting) ▪ Going to the bathroom, toilet paper rolls, touching one’s genital area (washing one’s entire body thoroughly), asking for nighttime diaper care after touching one’s genitals, putting on feces, less abnormal behavior when a woman takes interest and treats them, being cared for by the opposite sex	11 (10.7)
Occupation	▪ Walking exercise ▪ Hwatu (Korean traditional card game), puzzles, reading books, reading magazines (picture books), thinking of times when they spend money	7 (6.8)
Inclusion	▪ Conversation, talking, emotional support, taking their side to support, holding hands in conversation, warm conversation, face to face conversation, listening to what they want and appeasing them, staying with them, being a conversation partner while having their favorite snack, talking with them while meeting eyes, friendly and gentle approach, others answering well to the same questions, listening to complaints quietly ▪ Listening to positive expressions about own behavior, words or expressions of acknowledgment, emotional care (using cyclical language), attention from those that care for them and adaptation to the environment	25 (24.3)

*BPSD* behavioral and psychological symptoms of dementia

### Immersion, preference, and interaction during VR experience

All participants were female. The mean age of the participants was 85.80 ± 3.26 years (Min 82 ~ Max 90, Median [IQR] 85.5 [84.25–88.75]). The participants had a mean MMSE-K score of 21.44 ± 4.59 points (Median [IQR] 22 [18.00–24.00]) and a mean ADL score of 4.33 ± 2.88 points out of 30 points (Median [IQR] 5 [1.75–6.00]).

As the participants engaged with the VR program, the researchers observed the level of the participants’ immersion, preference, and interaction with the VR on a 5-point Likert scale. The mean immersion score was 4.93 ± 0.16 points (Median [IQR] 5.00 [4.87–5.00]), the mean preference score was 4.35 ± 0.41 points (Median [IQR] 4.65 [3.84–4.90]), and the mean interaction score was 3.84 ± 0.43 points (Median [IQR] 3.85 [3.58–4.30]) in each of the virtual places.

### Tolerance for VR experience

There were multiple indicators of VR tolerance. First, six out of the 10 participants required assistance, such as help with wearing the HMD, focusing their visual acuity on the HMD glasses, and capturing their hand movements via leap motion. The mean VR experience duration was 10.00 ± 3.46 min. The participants’ reactions throughout the VR were classified into positive or negative outcomes. Seven participants had positive responses to VR, while the remaining three had both positive and negative responses. The positive reactions included pleasure, such as focusing on the program for a set period, laughing, and communicating verbally and non-verbally in a stable state. Negative emotions were recorded as the occurrence of complaining (Table 3).

**Table 3:** Level of immersion, preference, interaction and tolerance of VR (N = 10)

No	Assistance device	Immersion		Preference		Interaction		Tolerance for VR			
		M ± SD	Min-max	M ± SD	Min-max	M ± SD	Min-max	Need assistance	Length of experience (min)	Participants reaction	VR sickness
1	Cane	5.00 ± 0.00	5	5.00 ± 0.00	5	4.63 ± 0.35	4–5	No	15	Positive	–
2	Cane	4.85 ± 0.38	4–5	4.85 ± 0.38	4 ~ 5	4.38 ± 0.45	4–5	No	15	Positive	–
3	–	5.00 ± 0.00	5	4.92 ± 0.29	4 ~ 5	4.64 ± 0.75	3–5	No	10	Positive	–
4	Walker	4.65 ± 0.24	4.5–5	3.55 ± 0.37	3 ~ 4	2.80 ± 0.76	2–3.5	Yes	10	Positive/ negative	+
5	Wheel chair	5.00 ± 0.00	5	2.88 ± 1.44	2 ~ 5	3.00 ± 1.47	1.5–5	Yes	5	Positive/ negative	–
6	Walker	4.94 ± 0.18	4.5–5	4.50 ± 0.71	3.5 ~ 5	3.56 ± 1.43	1–5	Yes	8	Positive	–
7	Wheel chair	5.00 ± 0.00	5	5.00 ± 0.00	5	3.63 ± 0.48	3–4	Yes	10	Positive	+
8	Walker	5.00 ± 0.00	5	3.70 ± 0.67	2.5–4	3.70 ± 0.67	3–4.5	Yes	5	Positive	–
9	Cane	5.00 ± 0.00	5	4.28 ± 0.51	3–4.5	4.00 ± 1.41	2–5	No	10	Positive/ negative	–
10	Wheel chair	4.83 ± 0.39	4–5	4.79 ± 0.40	4–5	4.07 ± 0.73	3–5	Yes	12	Positive	–
Mean ± SD		4.93 ± 0.16		4.35 ± 0.41		3.84 ± 0.43			10.00 ± 3.46		
Min-Max		4.65–5.00		2.88–5.00		2.80–4.64					
Median (IQR)		5.00 (4.87–5.00)		4.65 (3.84–4.90)		3.85 (3.58–4.30)					

*VR* virtual reality, *IQR* interquartile range

## Discussion

The purpose of this study was (1) to develop a VR intervention program based on the psychological needs of patients residing in South Korean nursing facilities, (2) to test whether this VR intervention alleviates BPSD, and (3) to confirm the utilization of VR in patients with dementia. This program aims to improve the patients’ quality of life and provide effective care models for health professionals.

The VR program was designed to meet the participants’ psychological needs by securing their emotional immersion and engagement in accordance with the preferences and emotions of South Koreans. Comfort needs were most frequently suggested as a way to alleviate the patients’ behavioral and psychological symptoms. Patients with dementia maintain their identity through love and comfort, which provides them with a greater sense of well-being [34, 35]. Patients with dementia obtain happiness and enjoyment by engaging in meaningful activities, such as various leisure activities, social participation, and work-related activities, which improve the individual’s sense of autonomy and identity [36, 37]. When their psychological needs were satisfied, their anxiety and behavioral psychological symptoms decreased [38, 39], and such reductions have been effective in reducing the need for neuroleptics [16]. Additionally, this VR environment provides a multimedia platform that can facilitate the storage and retrieval of memories and simulate multisensory experiences. This could be used to create a therapeutic environment for alleviating BPSD.

The VR content developed in this study was applied to patients with dementia, and the evaluations were subsequently obtained from the participants’ immersion, preference, interaction, and tolerance for VR. The participants’ feedback was consistent with previous results demonstrating that patients with dementia participated more keenly in activities that reflected their individual preferences [35] and activities that they could recall and reminisce about [40–43]. A high degree of preference and immersion were confirmed among the participants, demonstrating the usability of the VR intervention program. This indicates that it is important for VR platforms to present a familiar environment, given the historical and cultural backgrounds of patients with dementia. Furthermore, intervention programs that reflect familiarity and various emotional stimuli can increase immersion and contribute to active interactions by helping the participants reminisce and recall the past [44].

Manera et al. [15, 45] reported that older individuals with mild cognitive disorders or Alzheimer’s disease had higher levels of satisfaction and reported less anxiety, discomfort, and fatigue with image-based VR exercises compared to paper and pencil exercises. The results of these studies indicate that well-developed VR content can be effectively applied to patients with dementia and mild cognitive impairment and can result in positive outcomes [46].

Despite these promising results, the usability problems of the VR system developed in this study need to be solved. In terms of the experience length, this varied from 5 to 15 min. This was because the participants chose different themes, and their interaction pace varied. The theme “street of memories” was selected the most, which included an elementary school, an alleyway, and a homely house. The theater and coffee house were selected the least. Considering that the participants’ mean age was 85.80 years, watching movies in the theater and drinking coffee in a café might not be preferred activities for these participants. Therefore, historical and cultural characteristics should be considered to develop immersive VR programs [47].

In this feasibility study, there were issues related to the assistance required to use the VR: the weight of the HMD (Samsung HMD Odyssey Windows Mixed Reality Headset and Wireless controllers 630 g), controlling the visual acuity, and capturing the participants’ hand movements via leap motion. The participants displayed difficulties operating the VR with hand movements mostly likely because of the decreased hand movement and cognitive functions of the older patients [48]. The patients complained of limited vision while wearing the HMD as they are cut off from the outside world; they also complained about the heavy weight of the displays [20]. The weight of the HMD is 590 g, which is relatively lighter than other HMDs; even lighter HMDs for older patients will be released in the future (e.g., HP Reverb G2). The VR environment requires active movement and the use of visual and auditory senses. This may be limited in older people with dementia since they typically experience reduced visual and auditory capacity, resulting in decreased accuracy and attention [5]. The results of this study indicate that the participants experienced some difficulties due to their diminished sensory capacity. This should inform the design, implementation, and evaluation of similar technologies or interventions. For instance, physical functions, such as visual acuity and hand power, and the weight of the HMD should be considered for the use of VR technology with dementia patients.

In the current study, VR sickness varied with two participants complaining about dizziness or nausea during the VR immersion. The VR intervention program test was implemented while the participants were seated, which decreased the effort required to maintain their posture. Clearer images and accurate motion tracking can reduce VR sickness [49, 50], and content requiring less movement [51] is less likely to lead to VR sickness. Although these symptoms are temporary [52], researchers and developers need to pay attention to this issue.

This feasibility study aimed to uncover the strengths and weaknesses of VR interventions. The findings demonstrate that it is feasible to use VR with patients with dementia but did not demonstrate the efficacy of VR to reduce BPSD. Further research should be conducted based on rigorous experimental design to build more evidence for VR intervention programs. High-quality methodological and experimental studies are required to demonstrate the effectiveness of randomized controlled trials and should be designed to include a control group of dementia patients as a long-term intervention program in a nursing home.

Typically, VR platforms have been underutilized in healthcare services for groups such as patients with chronic diseases or people with disabilities [53]. This is the first study to develop VR interventions for dementia patients by considering their psychological needs. Overall, this study has demonstrated that VR could increase immersion, preference, interaction, and tolerance, but such effects vary with the nature of the task. Moreover, unlike previous studies, this study demonstrated that a low demand VR environment that facilitates the storage and retrieval of preferred memories could permit the implementation of strategies to facilitate patients’ interactions. While VR-based programs have issues, such as high costs, difficulty in operating the system, space constraints, and low portability [54, 55], the VR program employed in this study increased the convenience and safety of use by using simple interactive motions, such as pointing one’s hand in the desired direction and avoiding the need for large and complex hardware.

### Limitations and implications of the study

This study was a feasibility test with a limited sample. Future studies should recruit a large sample of participants and consider factors such as age, gender, personality type, and severity of dementia. Additionally, we used the trained researchers’ observations to screen the negative and positive responses during VR experience among the participants. The participants’ minor discomfort could not be reported because we did not use a standard measure. Researchers should continue to explore the potential discomfort of VR or sickness related to VR among the participants with cognitive decline using various measures such as self-reported questionnaires and standardized observations. In this study, there may have been some response bias since the researchers and assessors were the same. After completing the intervention, collecting immediate feedback from participants with moderate-to-severe dementia can be challenging since they struggle to respond to these types of questions. A mixed-methods approach involving semi-structured interviews and observational data should be used to assess their responses in future studies.

However, the VR-based intervention program developed in this study to reduce BPSD was feasible for the participants and provided them with a relatively high degree of satisfaction and immersion. Furthermore, this study also confirmed the convenience and safety of the program. These findings build evidence for the potential utility of VR programs to alleviate BPSD.

## Supplementary Information

**Additional file 1.** Open-ended questions for primary caregivers; Observation and records for patients with MCI or Dementia.

**Additional file 2.** Observation and records for patients with MCI or Dementia.

## Data Availability

The dataset used and analyzed during the current study is available from the corresponding author on reasonable request.

## Abbreviations

BPSD: Behavioral and psychological symptoms of dementia
VR: Virtual reality
MMSE-K: Mini mental status examination-korean version
ADL: Activities of daily life
HMD: Head mounted display
