# Supplementary material for: Developing a virtual reality for people with dementia in nursing homes based on their psychological needs: a feasibility study
Source: BMC Geriatr. 2021 Mar 7;21:167. doi: 10.1186/s12877-021-02125-w (PMC7938563; doi:10.1186/s12877-021-02125-w)
Supplement: Supplementary file 1 — Additional file 1. Open-ended questions for primary caregivers; Observation and records for patients with MCI or Dementia. [file 12877_2021_2125_MOESM1_ESM.docx]

Phase 1.

Open-ended questions for participants

“When you are feel agitation, aggression, psychosis, depression and apathy, what activities make you feel better or relieve the BPSD of you?

Answer _______________________________

If the participant could not answer the question

“What activities relieve the BPSD of the patient for whom you are caring?”

Answer _______________________________
