# Supplementary material for: Developing a virtual reality for people with dementia in nursing homes based on their psychological needs: a feasibility study
Source: BMC Geriatr. 2021 Mar 7;21:167. doi: 10.1186/s12877-021-02125-w (PMC7938563; doi:10.1186/s12877-021-02125-w)
Supplement: Supplementary file 2 — Additional file 2. Observation and records for patients with MCI or Dementia. [file 12877_2021_2125_MOESM2_ESM.docx]

Phase 3.

Observation and records for patients with MCI or Dementia

■ Medical records

1. Age ( )

2. Cognitive function: MMSE-K ( ) or K-MMSE ( )

3. Clinical dementia rating stage: CDR ( )

4. Ability to perform daily activities: ADL ( )

■ Level of immersion, preference and interaction during experience of VR-based intervention programs (5-point Likert scale)

| Scenario title | Places | Immersion  (1~5) | Preference  (1~5) | Interaction  (1~5) |
| --- | --- | --- | --- | --- |
| Train of memories | Train station |  |  |  |
| Streets of memory | Elementary school |  |  |  |
|  | Neighborhood alleys |  |  |  |
|  | Old houses |  |  |  |
| Nostalgic youth | Theater |  |  |  |
|  | Traditional coffee house |  |  |  |
|  | Traditional market |  |  |  |
|  | Old houses |  |  |  |
| Homely hometown | Valley |  |  |  |
|  | Reeds field |  |  |  |
|  | Rural home |  |  |  |
| Where I want to go | Orchards |  |  |  |
|  | Namiseom Island |  |  |  |
|  | The sea at night |  |  |  |
|  | Crocks of condiments |  |  |  |
|  | Jungnogwon bamboo garden |  |  |  |

5-point Likert scale: 1= “Very poor” 2= “Poor” 3= “Fair” 4= “Good” 5= “Excellent”

■ Participants’ tolerance for VR.

1. Need assistance to utilize VR: Yes ( ) No ( )

2. Duration of VR experience: ( )minutes

3. Patients reaction during VR experience _________________________

4. VR sickness: Yes ( ) No ( )
